# Inhibition of polo-like kinase 4 (PLK4): a new therapeutic option for rhabdoid tumors and pediatric medulloblastoma

**DOI:** 10.18632/oncotarget.22704

**Published:** 2017-11-24

**Authors:** Simone Treiger Sredni, Anders W. Bailey, Amreena Suri, Rintaro Hashizume, Xingyao He, Nundia Louis, Tufan Gokirmak, David R. Piper, Daniel M. Watterson, Tadanori Tomita

**Affiliations:** ^1^ Ann and Robert H. Lurie Children’s Hospital of Chicago, Division of Pediatric Neurosurgery, Chicago, IL 60611, USA; ^2^ Northwestern University, Feinberg School of Medicine, Department of Surgery, Chicago, IL 60611, USA; ^3^ Stanley Manne Children’s Research Institute, Cancer Biology and Epigenomics, Chicago, IL 60614, USA; ^4^ Northwestern University, Feinberg School of Medicine, Department of Neurological Surgery, Chicago, IL 60611, USA; ^5^ Thermo Fisher Scientific, Research and Development, Biosciences Division, Carlsbad, CA 92008, USA; ^6^ Northwestern University, Feinberg School of Medicine, Department of Pharmacology, Chicago, IL 60611, USA

**Keywords:** embryonal tumors, CNS, AT/RT, RTK, polyploidy

## Abstract

Rhabdoid tumors (RT) are highly aggressive and vastly unresponsive embryonal tumors. They are the most common malignant CNS tumors in infants below 6 months of age. Medulloblastomas (MB) are embryonal tumors that arise in the cerebellum and are the most frequent pediatric malignant brain tumors. Despite the advances in recent years, especially for the most favorable molecular subtypes of MB, the prognosis of patients with embryonal tumors remains modest with treatment related toxicity dreadfully high. Therefore, new targeted therapies are needed.

The polo-like kinase 4 (PLK4) is a critical regulator of centriole duplication and consequently, mitotic progression. We previously established that PLK4 is overexpressed in RT and MB. We also demonstrated that inhibiting PLK4 with a small molecule inhibitor resulted in impairment of proliferation, survival, migration and invasion of RT cells.

Here, we showed in MB the same effects that we previously described for RT. We also demonstrated that PLK4 inhibition induced apoptosis, senescence and polyploidy in RT and MB cells, thereby increasing the susceptibility of cancer cells to DNA-damaging agents. In order to test the hypothesis that PLK4 is a CNS druggable target, we demonstrated efficacy with oral administration to an orthotropic xenograft model.

Based on these results, we postulate that targeting PLK4 with small-molecule inhibitors could be a novel strategy for the treatment of RT and MB and that PLK4 inhibitors (PLK4i) might be promising agents to be used solo or in combination with cytotoxic agents.

## INTRODUCTION

Rhabdoid tumors (RT), or malignant rhabdoid tumors (MRT), are among the most aggressive and lethal forms of human cancer. They can arise in any location in the body but are most commonly observed in the central nervous system (CNS), where they are called atypical teratoid/rhabdoid tumors (AT/RT). When arising in the kidneys they are called rhabdoid tumors of the kidney (RTK) while extracranial extrarenal rhabdoid tumors are generically called eMRT. Independent of their site of origin, RT are recognized as the same entity [1] with the vast majority showing loss of function of the *SMARCB1* gene or, to a lesser extent, the S*MARCA4* gene, both members of the SWI/SNF chromatin-remodeling complex [2]. RT occurs predominantly in infants and children less than 3 years of age and although considered to be rare, AT/RT is the most common malignant tumor of infants below 6 months of age [3]. The overall survival is poor with median survival around 17 months [4]. Introduction of anthracycline-containing chemotherapy regimens resulted in survival improvement, however with significant morbidity [5]. Radiation is also an effective component of therapy but needs to be avoided in patients younger than 3 years of age due to long term neurocognitive sequelae. Recently, investigations of altered signaling pathways have yielded a whole array of compounds with potential therapeutic activity, some of which are currently in clinical trials, including AURKA, EZH2 and CDK4/6 inhibitors [3]. However, despite the advances in recent years, the overall survival of these young patients remains poor and treatment related toxicity, high.

Medulloblastoma (MB) is an embryonal tumor of the cerebellum which is the most common malignant brain tumor in children and a major cause of mortality in pediatric oncology. Molecular studies from several groups around the world demonstrated that MB consists of four distinct molecular subgroups: WNT, Sonic Hedgehog (SHH), group 3, and group 4. Each subgroup differs in demographics, transcriptomes, somatic genetic events, and clinical outcomes [6, 7]. Regardless, current therapies for MB consist mostly of cytotoxic agents and mortality is still significant, with survivors exhibiting treatment-related effects as a consequence of cytotoxic chemotherapy and radiation [8].

Clearly, new targeted therapies are urgently needed. Our long-term goal is to identify new, more effective and less toxic anticancer therapies for RT and other pediatric embryonal tumors. In this regard, we previously demonstrated that RT cell proliferation is dependent on PLK4 and suggested that PLK4 is a candidate target for the treatment of RT and possibly other embryonal tumors. We accomplished this by performing a functional screening of the kinome to investigate essential kinases for RT proliferation. We used lentiviral CRISPR/Cas9 particles (Invitrogen™ LentiArray™ CRISPR Libraries, Thermo Fisher Scientific, USA) to individually mutate 160 kinases representing every major branch of the kinome. Mutations in the polo-like kinase 4 (*PLK4*) gene resulted in the most significant impairment of RT cell proliferation. We also established that the PLK4 inhibitor (PLK4i) CFI-400945 had *in vitro* therapeutic effects on RT cells [9] and detected PLK4 overexpression in pediatric MB [10].

The drug candidate CFI-400945 used in our previous studies is an effective PLK4 inhibitor [11, 12] and recently entered a phase I clinical trial to establish its safety, tolerability and pharmacokinetics in advanced solid tumors in adults (NCT01954316). Preliminary results indicated that the drug is “well tolerated at doses up to 72 mg and has a favorable PK profile” [13]. PLK4 plays a key role in cell cycle control. It localizes to the centrosomes, being a critical regulator of centriole duplication and consequently, mitotic progression [14–17]. The proposed role of PLK4 in the regulation of cytokinesis and maintenance of chromosomal stability is consistent with a function in cancer, as centrosome amplification can drive genetic instability with a resultant impact on tumorigenesis. Consistent with our results in RT cells, PLK4 is overexpressed in human gastric [18], breast [11] and pancreatic cancer [19]. Therefore, there is an evolving trend of PLK4 up-regulation in diverse cancers and promising responses to treatment of such tumors with PLK4 inhibitor drug candidates such as CFI-400945. However, little is known about CFI-400945 in CNS tumors. Further, its molecular properties place it at the cusp of key multi-property profiles that characterize drugs with known *in vivo* blood brain barrier (BBB) penetrance sufficient for brain target engagement [20]. Therefore, we explored the potential of CFI-400945 to be an *in vivo* candidate for brain tumors such as AT/RT and gain insight into its molecular selectivity in the kinome target family.

We report here that MB cell lines derived from molecular subgroups with the most aggressive behavior (DAOY – from sonic hedgehog and D283 – from groups 3/4) [21] were susceptible to the PLK4i CFI-400945 which impaired cell proliferation, cell viability, colony formation, migration and invasion, and induced polyploidy and senescence in these cells. We further illustrate the mechanism of action of the PLK4 inhibitor in these embryonal tumors. In order to extend our hypothesis to the *in vivo* context, we examined the efficacy of CFI-400945 in orthotropic AT/RT xenografts. Based on the results, we conclude that this PLK4 inhibitor can increase the susceptibility of embryonal cancer cells to DNA-damaging agents due to its role in the initiation and maintenance of polyploidy. Further, we suggest that the next generation of brain tumor focused PLK4 inhibitors could be promising agents for use in combination therapies for the treatment of RT and MB.

## RESULTS

### Medulloblastoma (MB) cells were susceptible to the therapeutic effects of the PLK4 inhibitor

**Figure 1:**
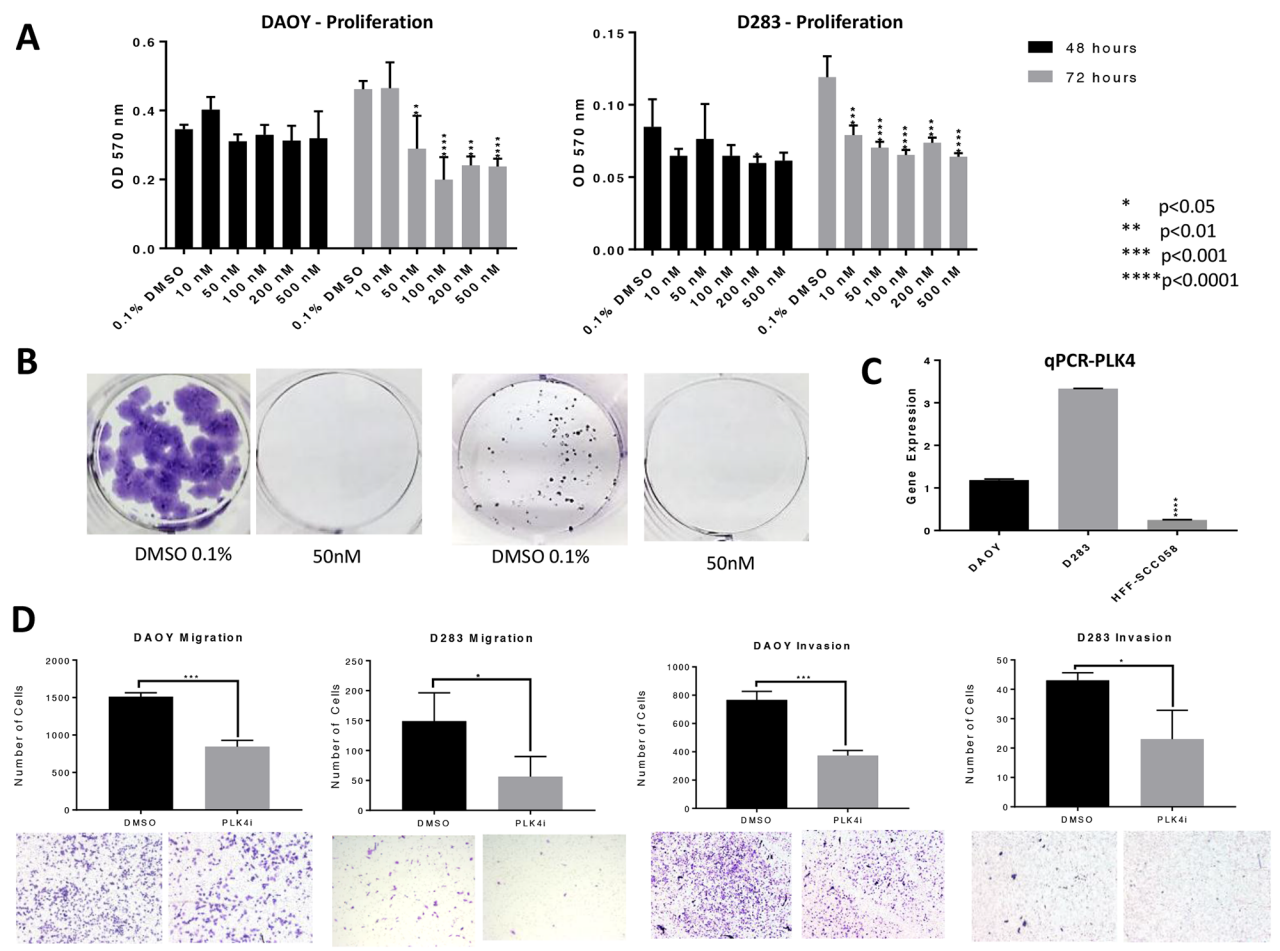
PLK4 is overexpressed in MB and CFI-400945 significantly affected MB cell lines **(A)** MTT proliferation assays – CFI-400945 significantly decreased proliferation in both cell lines compared to the control (0.1% DMSO) after 72 hours of treatment. **(B)** Complete inhibition of colony formation was observed at a concentration of 50nM of the PLK4i in DAOY and D283 respectively. **(C)** qRT-PCR showed significantly higher expression of *PLK4* in DAOY and D283 when compared to HFF-SCC058 human fibroblasts. **(D)** Cell migration and invasion were assessed after 24 hour treatment with 100nM PLK4i. Both the DAOY and D283 treated cell lines exhibited significant decrease in cell migration and invasion compared to control (0.1% DMSO).

**Figure 2:**
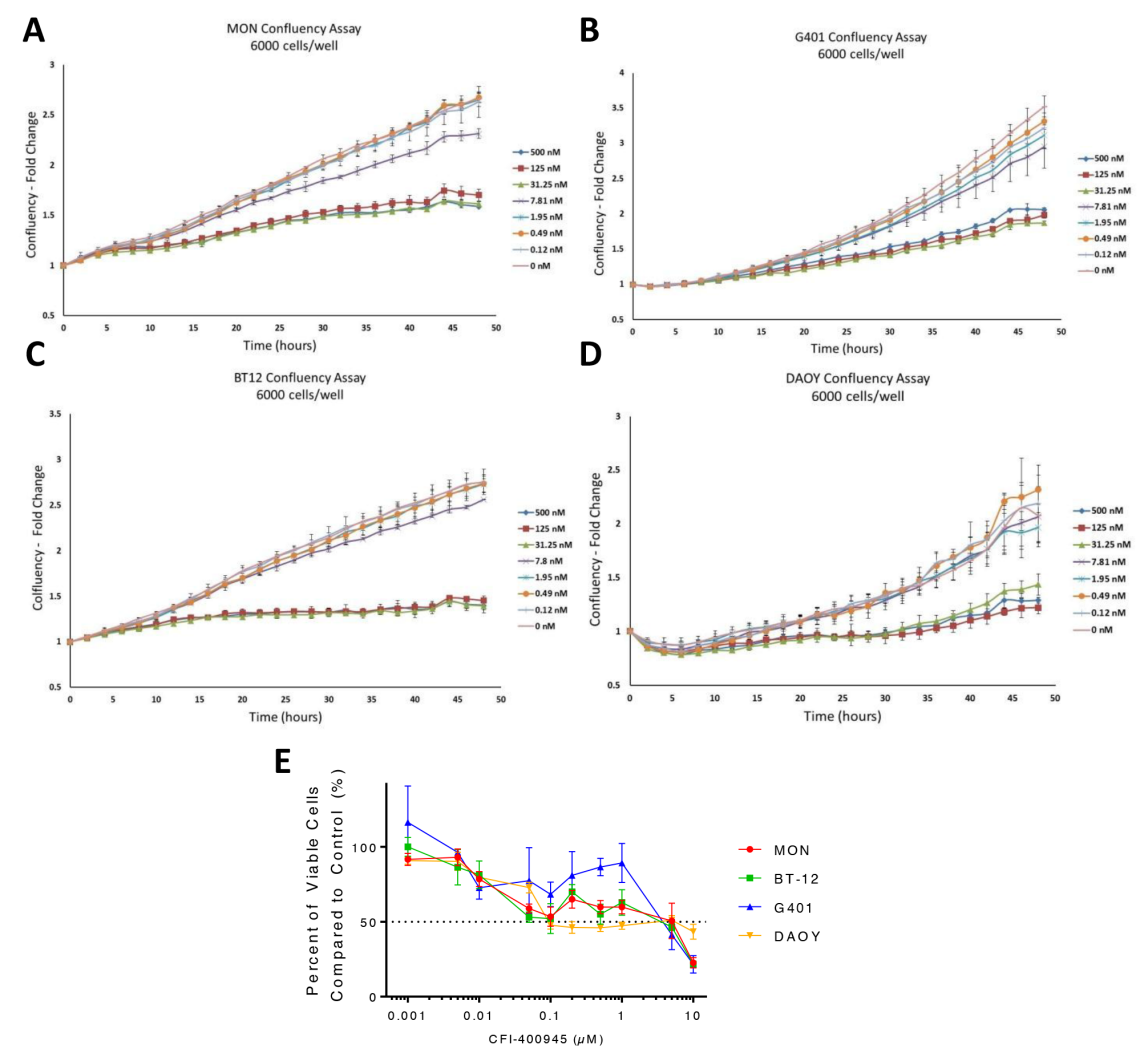
CFI-400945 affected tumor cell ability to reach confluency and had an impact on cell viability Significant delay in reaching confluency was observed when MON, G401, BT-12 and DAOY cells were treated with increasing concentrations of CFI-400945 starting at 31.25nM. Cells were plated at a density of 6,000 cells/well. **(A)** MON MRT cells (FC=1.56, p-value=0.004); **(B)** G401 RTK cells (FC=1.78, p-value=0.004); **(C)** BT-12 AT/RT cells (FC=1.89, p-value=0.001) and **(D)** DAOY MB cells (FC=1.70, p-value=0.029); **(E)** Dose-response curves created from viability assays for three rhabdoid cell lines and one MB cell line treated with concentrations of CFI-400945 ranging from 1nM to 10μM. IC50 values were calculated: MON (5.13μM), BT-12 (3.73μM), G401 (3.79μM), DAOY (0.094μM).

We previously demonstrated that PLK4 was overexpressed in RT and other embryonal tumors of the brain, including MB [9, 10]. We then demonstrated that RT cell proliferation is dependent on PLK4 and that PLK4 inhibitors may represent a novel therapeutic approach for these aggressive and unresponsive tumors. Now, we demonstrated that the MB cell lines DAOY and D283, representative of the most aggressive MB molecular subtypes (sonic hedgehog and groups 3/4, respectively) [21] are also susceptible to the PLK4i CFI-400945 (Figure 1). Then, we provided additional evidence of the potential therapeutic effects of CFI-400945 by demonstrating its effect on cell viability and the rate of the cells to reach confluency in 4 different cell lines, including RT originating in different locations (MON-soft tissue, G401-kidney, BT-12-brain) and in the DAOY MB cell lines (Figure 2).

### The PLK4 inhibitor CFI-400945 is a multi-kinase inhibitor with a defined selectivity

Kinase screening profile revealed 10 kinases, including PLK4, in which activity was inhibited above 80% by CFI-400945. IC50 values were determined by a 10-point titration curve analysis. The inhibited kinases within these parameters were: PLK4 – 100%, IC50 4.85nM; AURKB – 95%, IC50 70.7nM; AURKC – 84%, IC50 106nM; DDR2 – 100%, IC50 315nM; MUSK – 96%, IC50 48.7nM; NTRK1 – 92%, IC50 20.6nM; NTRK2 – 96%, IC50 10.6nM; NTRK3 – 96%, IC50 9.04nM; ROS1 – 98% and TEK – 96%, IC50 109nM. The activity of aurora kinase A was inhibited by 60% (AURKA – 60%, IC50 188nM) (Supplementary Table 1). CFI-400945 has a defined set of potential kinase targets that are logical contributors to its known phenotypic effects, with PLK4 in the higher affinity group, consistent with the prevailing view that kinase targets with IC50 < 1,000 nM are likely *in vivo* targets and a selective multi-kinase profile is needed for efficacy [22].

### Key features of the PLK4 inhibitor CFI-400945 suggest a potential for lower cardio toxicity or drug-drug interaction risk

**Figure 3:**
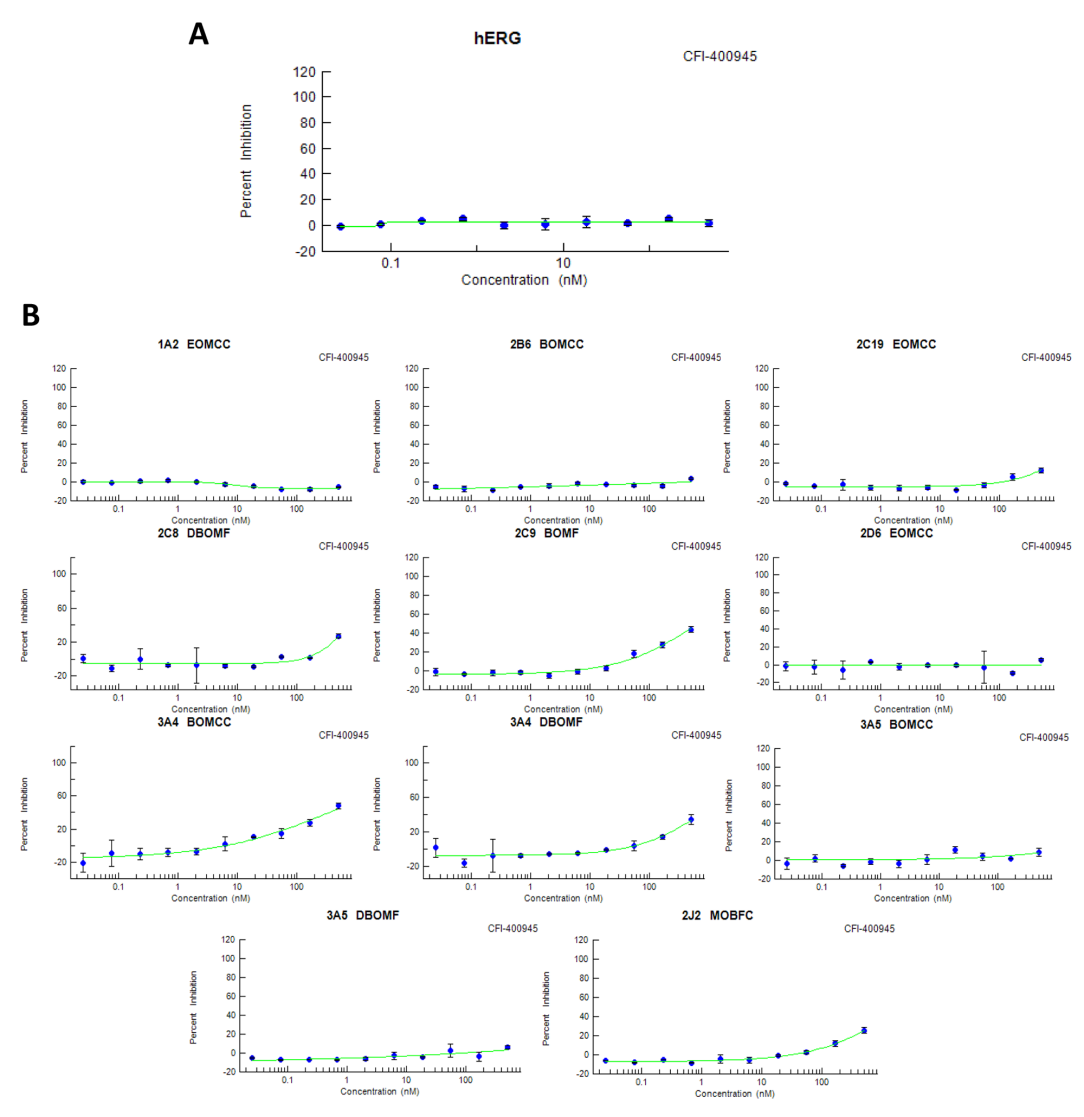
CFI-400945 – Drug safety and toxicology **(A)** CFI-400945 did not bind to the hERG K^+^ channel (IC50>500nM); **(B)** CFI-400945 showed IC50 ≥ 500nM (the highest concentration tested) in screens, with positive controls, for each isoform considered of potential risk, except for 3A4 BOMCC which had an IC50 of 413nM (also negative based on the 3.16nM for the positive control ketoconazole).

Screening of drug candidates for hERG potassium channel inhibition has become an early step in testing for potential drug dependent long QT syndrome that is linked to sudden death [23]. Binding of CFI-400945 at the hERG channel was not detected under standard assay procedures at the highest concentration tested (IC50>500nM), while the IC50 value for the positive control E-4031 was 35.1nM (Figure 3A). These data suggest that CFI-400945 is not in the high risk category for cardiovascular risk, although the FDA recommended *in vivo* cardiovascular toxicology would be required for investigational new drug status.

Cytochrome P450 (CYP450) enzymes are drivers of first-pass metabolism for orally administered drugs, with certain CYPs being known pharmacogenetic contributors to individual variance in drug safety and efficacy and others contributing to undesired drug-drug or drug-food interactions, that can contribute to toxicities or therapeutic failures [24]. Although there are more than 50 CYP isoenzymes, 90% of drugs are metabolized by 6 isoenzymes with the two of the most significant in risk being CYP3A4 and CYP2D6. CFI-400945 showed an IC50 value >500nM (the highest tested concentration) for the isoenzymes recommended for testing by FDA guidance except for 3A4 BOMCC, which had an IC50 of 413 nM in contrast to 3.16nM for the CYP3A4 canonical control substrate ketoconazole (positive controls were used for each isoenzyme tested) (Figure 3B). These results forecast lower risk category for multi-drug treatments in experiments or patients.

### Inhibition of PLK4 resulted in abnormalities of centriole duplication and transcript expression

**Figure 4:**
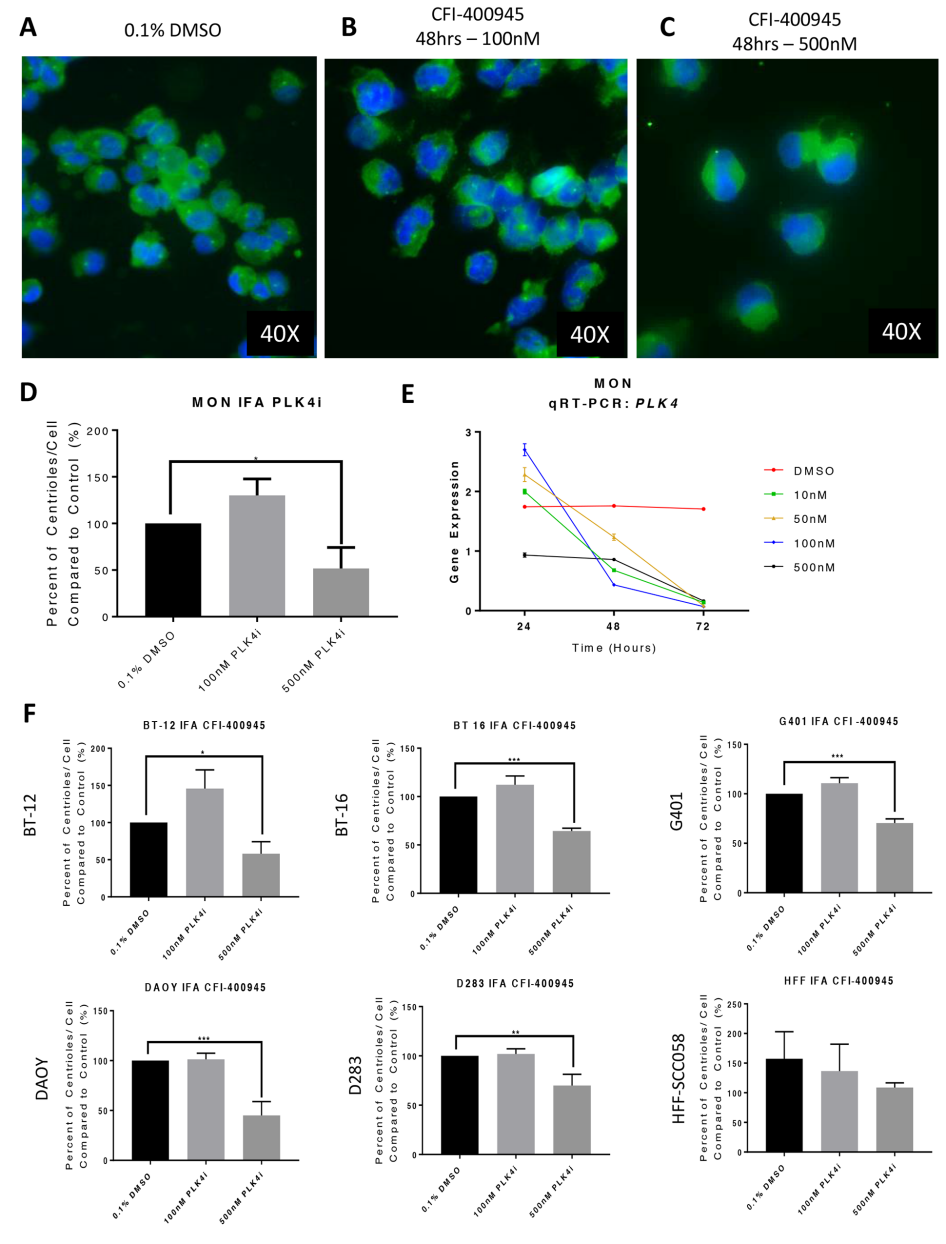
The *PLK4* inhibitor CFI-400945 affected centriole duplication and *PLK4* mRNA expression Immunofluorescence (IFA) for gamma-tubulin of MON rhabdoid cells treated with CFI-400945 for 48 hours (blue - nuclei, bright green – centriole). **(A)** Control – Cells treated with 0.1% DMSO. **(B)** When treated with 100nM there was a slight increase in centrioles’ number. **(C)** When treated with 500nM there was significant depletion of centrioles. **(D)** The ratio of number of centrioles/cell illustrates the “paradoxical effect”. **(E)** qPCR for *PLK4* in MON rhabdoid cells treated with 10, 50, 100 and 500nM CFI-400945 for 24, 48 and 72 hours demonstrated a time dependent decrease in *PLK4* expression compared to the control (0.1% DMSO). Note, that at 24 hours, the expression of the transcript increased at 10, 50 and 100nM, but not at 500nM, correlating with the increase in centrioles’ number. After 24hrs of exposure to the inhibitor, the mRNA expression decreased progressively. **(F)** The rhabdoid tumor (G401, BT-12 and BT-16) and the MB (DAOY and D283) cell lines showed increase in centriole’s number when treated with 100nM of the PLK4i suggesting increase in PLK4 expression, whereas a significant decrease is observed with 500nM. This “paradoxical effect” was not observed in the non-neoplastic human fibroblast cells (HFF-SCC058), which do not overexpress PLK4 (Figure 1C).

The MON rhabdoid tumor cell line treated with the PLK4i CFI-400945 demonstrated abnormalities in centriole duplication. An increase in centriole numbers was observed when treating each cell line with a lower concentration (100nM) for 48 hours, as demonstrated by immunofluorescence for gamma-tubulin. At a higher concentration of the PLK4i (500nM), we observed significant decrease in centriole number. A decrease in *PLK4* transcript expression was also detected by qRT-PCR in MON cells treated with increasing concentrations of the drug over time. A similar response was observed in additional RT and MB cell lines, but not in non-neoplastic human fibroblast that do not overexpress PLK4 [9] (Figure 4).

### Cells treated with the PLK4 inhibitor underwent apoptosis or senescence

**Figure 5:**
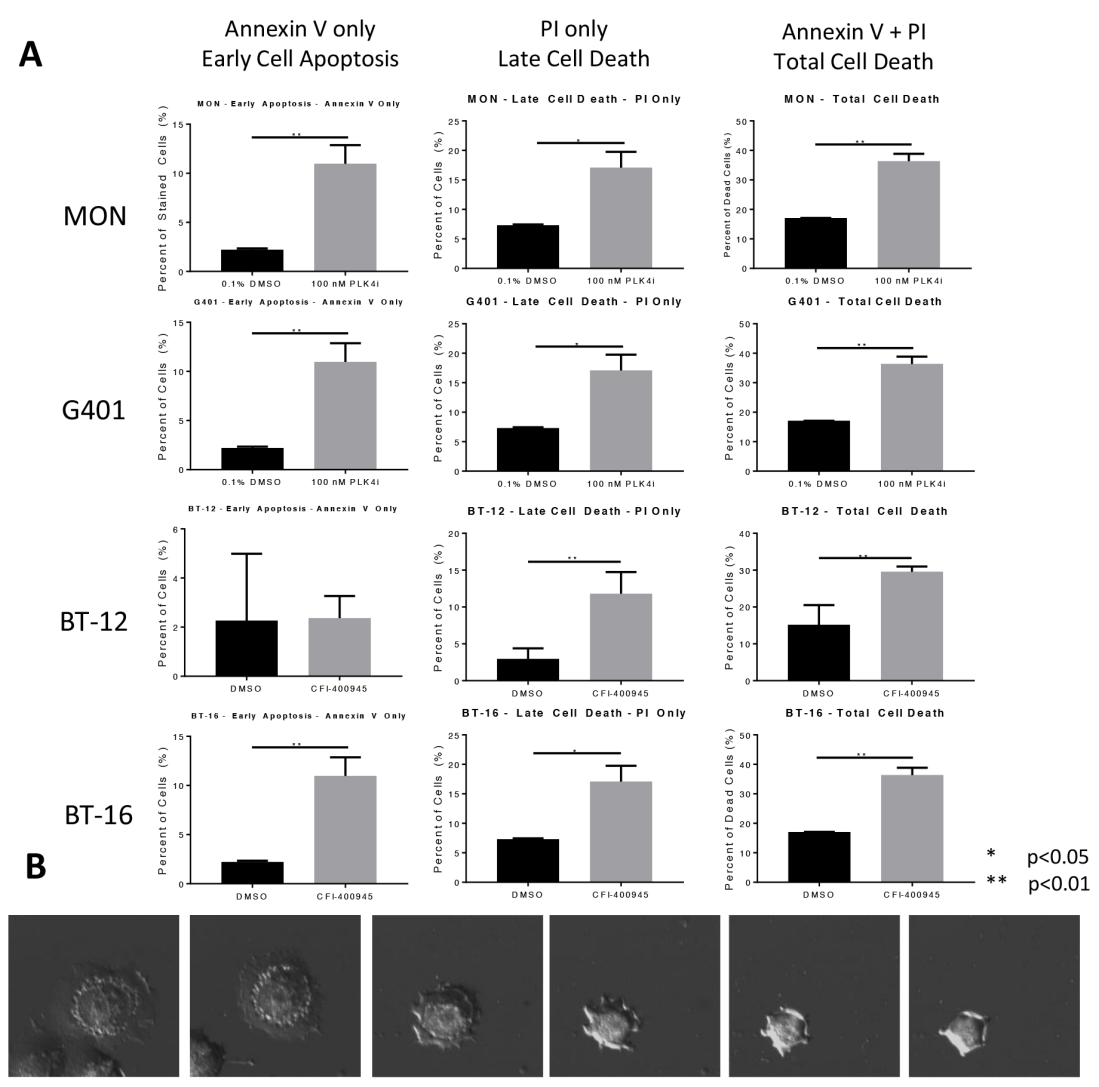
CFI-400945 induced cell death in multiple pediatric rhabdoid cell lines **(A)** Apoptosis was evaluated in four rhabdoid cell lines (MON, G401, BT-12 and BT-16) treated with 100nM CFI-400945 for 48 hours and measured by flow cytometry. A significantly higher percentage of cells undergoing cell death in the initial 48 hours of treatment, was observed when compared to the control (0.1% DMSO). **(B)** Images of a cell undergoing apoptosis within 48 hours of treatment with 100nM CFI-400945.

Rhabdoid tumor cells were evaluated for the impact of CFI-400945 on the induction of apoptosis by Annexin V staining. We observed a significant increase in total cell death in all rhabdoid cells investigated in the first 48 hours of exposure to the compound (100nM) as demonstrated in Figure 5. The mechanism for cell death at short time exposure to the compound is still to be elucidated.

**Figure 6:**
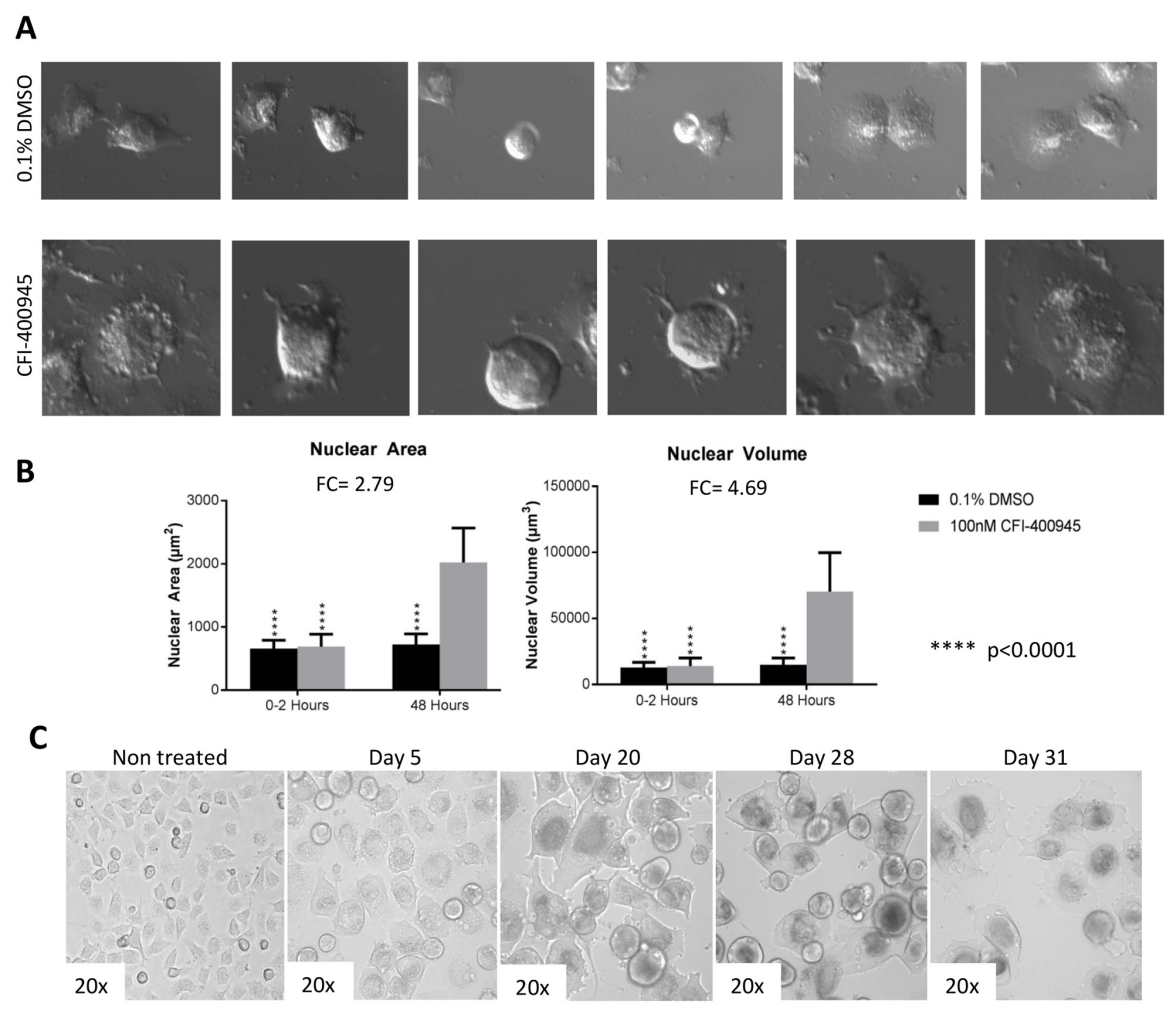
Rhabdoid cells treated with the PLK4 inhibitor CFI-400945 displayed significant increase in cell size (hypertrophy) **(A)** Images of MON cells treated with 100nM CFI-400945 or 0.1% DMSO (control) taken from the time-lapse video. Cells treated with 0.1% DMSO were observed to detach from the bottom of the dish and divide with two daughter cells reattaching to the plate. Cells treated with 100nM CFI-400945 were observed to detach from the plate and then reattach without dividing as much larger, single cells. These images illustrate the phenomenon of DNA endoreduplication without cytokinesis secondary to the depletion of centrioles as an effect of PLK4 inhibition. **(B)** The area and volume of the nuclei were measured for both the 0.1% DMSO (control) and 100nM CFI-400945 treated cells within the first two hours and at 48 hours of treatment. A significant difference was observed for both area (FC = 2.79; p-value<0.001) and volume (FC = 4.69; p-value<0.001) after treatment. **(C)** Images of MON cells treated with 100nM CFI-400945 for over 30 days displayed progressive cellular enlargement, culminating with cell death.

**Figure 7:**
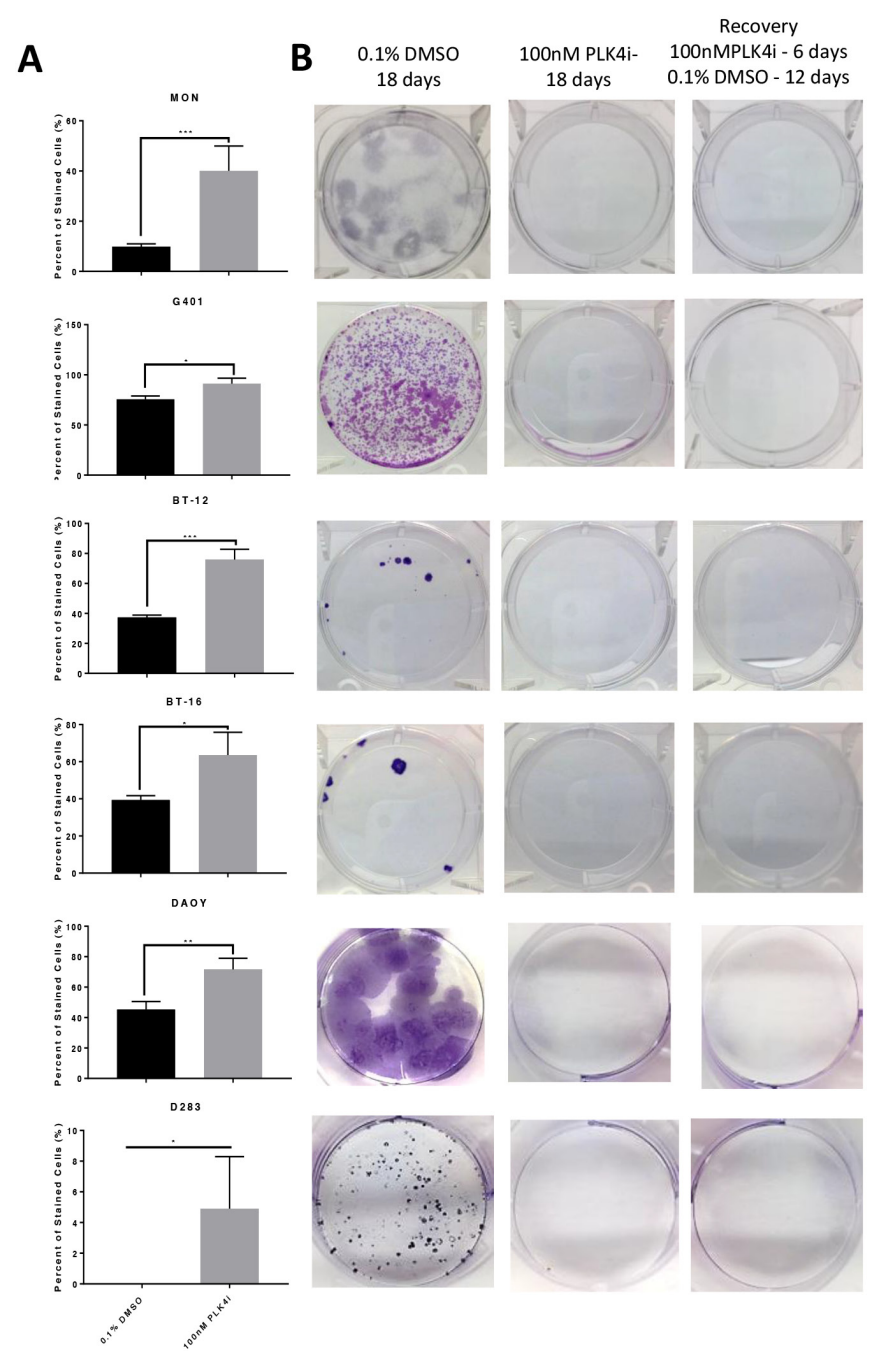
RT and MB cells exposed to the PLK4 inhibitor CFI-400945 underwent an irreversible state of cell cycle arrest - senescence **(A)** Beta galactosidase assay showed significant increase of senescent cells at 48 hours of CFI-400945 exposure (100nM) in all cell lines (MON, G401, BT-12, BT-16, DAOY and D283). **(B)** Clonogenic recovery assay: cells were incubated for 6 days with 100nM of CFI-400945, washed and exposed to 0.1% DMSO for an additional 12 days allowing for recovery. Control cells were incubated with 0.1% DMSO for 18 days or CFI-400945 100nM for 18 days. After 6 days of exposure to the drug, none of the cell lines recovered their ability to generate colonies further substantiating irreversibility of proliferation arrest.

Senescence is an irreversible state of complete cessation of cell division [25]. Observation of cells exposed to CFI-400945 (100nM) for an extended period of time (over 30 days) showed a progressive increase in cell size (hypertrophy), which is a sign of senescence [26, 27] (Figure 6). We also measured senescence via a beta-galactosidase assay, the current gold standard marker of senescence [28], and demonstrated significantly higher levels of senescence in treated cells when compared to the controls. The ability of a cell to re-enter into the cell cycle was evaluated by a clonogenic recovery assay. All cell lines exposed to the compound for 6 days lost their ability to generate colonies (Figure 7).

We performed a 48 hour time-lapse experiment to compare treated MON cells (100nM) to control MON cells exposed to 0.1% DMSO and witnessed that cells treated with the compound demonstrated inability to go through cytokinesis after DNA endoreduplication. As a result of abortive mitoses, an increase in nuclear volume and consequently, cell size was observed [27].

### Inhibition of PLK4 caused polyploidy in tumor cells while not affecting non-neoplastic human fibroblasts

**Figure 8:**
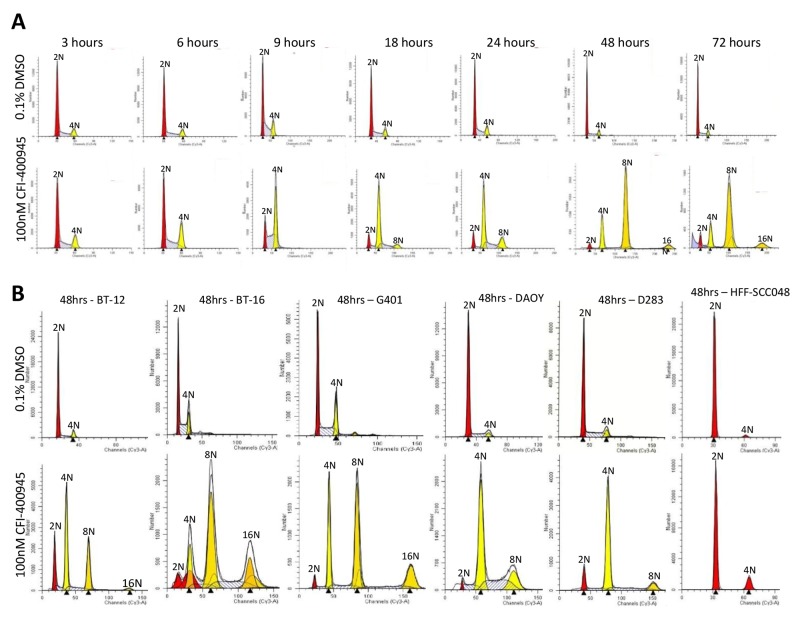
CFI-400945 induced polyploidy in multiple RT and MB cell lines, while sparing non-neoplastic human fibroblasts **(A)** Flow Cytometry Cell Cycle analysis of MON rhabdoid cells exposed to 100nM CFI-400945 for 3-72 hours revealed a time-dependent induction of polyploidy compared to the control (0.1% DMSO). **(B)** Flow Cytometry Cell Cycle analysis of G401 (RTK), BT-16 (AT/RT) and two MB cell lines (DAOY and D283) treated with 100nM CFI-400945 for 48 hours also displayed extreme polyploidy. No effect was observed in the non-neoplastic human fibroblast cell line HFF-SCC058 (that do not overexpress PLK4), when treated with 100nM of CFI-400945.

As a result of the inhibition of centriole duplication by the drug, the cells did not undertake cytokinesis becoming polyploid, as determined through cell cycle analysis by flow cytometry. Conversely, the non-neoplastic human fibroblast cell line HFF-SCC058, which has low expression of PLK4 [9], maintained its diploid status when challenged with CFI-400945 (Figure 8).

### Polyploidy induced by PLK4 inhibition sensitized RT and MB cells to cytotoxic effects of DNA-damaging drugs

**Figure 9:**
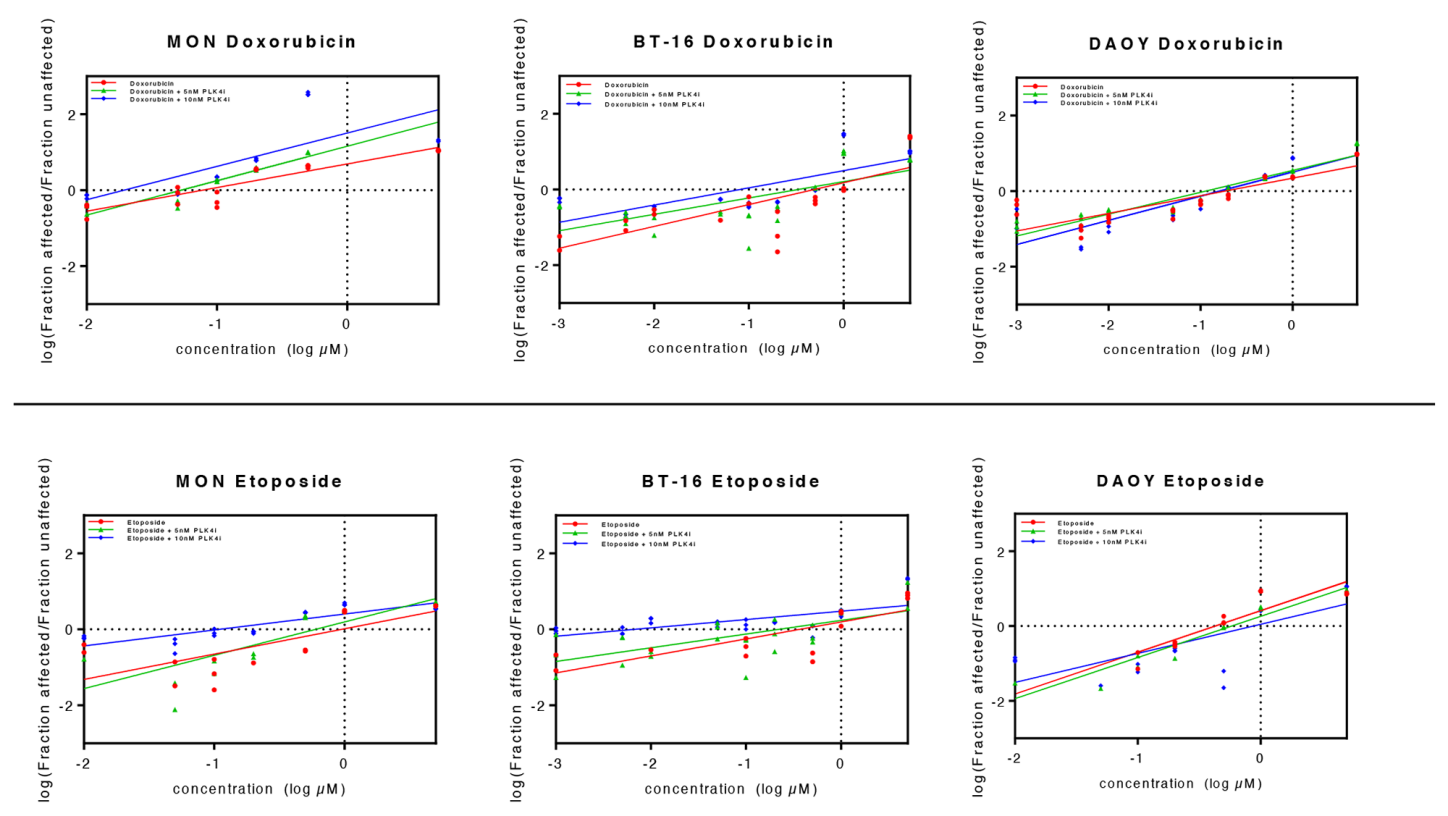
Association of CFI-400945 with conventional cytotoxic drugs doxorubicin and etoposide synergistically reduced viability of RT and MB cell lines 5nM and 10nM CFI-400945 were used in combination with doxorubicin and etoposide in concentrations ranging from 0.001 to 5μM. Viability was assessed comparing the effects of combination therapy versus treatment with each cytotoxic drug alone. Median effect plots showed that CFI-400945 in association with etoposide or doxorubicin significantly decreased the concentration of cytotoxic drug needed to affect viability of RT and MB cells (for IC50 values, please refer to Table 1).

We demonstrated that treatment of RT and MB cells lines with CFI-400945 inhibited PLK4 expression, inducing polyploidy as illustrated in Figure 8. Because it has been previously postulated that polyploidization increases the sensitivity of mammalian cells to DNA-damaging agents [29], and it is well known that doxorubicin exhibits clinically relevant cytotoxicity in RT [30–32], we hypothesized that combining CFI-400945 with conventional chemotherapy drugs like doxorubicin and etoposide would act synergistically on the induction of apoptosis. Using a viability assay, we demonstrated that cells simultaneously treated with the PLK4 inhibitor and doxorubicin or etoposide exhibited significantly lower IC50 values than cells receiving monotherapy at equivalent doses, providing evidence that cells became significantly more sensitive to the cytotoxic effects of doxorubicin and/or etoposide when treated in association with the PLK4 inhibitor (Supplementary Table 1 and Figure 9).

**Table 1:** Association therapy of CFI-400945 with DNA-damaging drugs as determined by a viability assay

Cell Line	Compound 1	Compound 2	IC50 of compound 2 (μM)	Standard deviation compound 2	P value^*^
MON		Doxorubicin	0.134	0.01713	
MON	CFI-400945 (5nM)	Doxorubicin	0.074	0.00125	0.0631
MON	CFI-400945 (10nM)	Doxorubicin	0.057	0.00806	0.0386
MON		Etoposide	0.967	0.00668	
MON	CFI-400945 (5nM)	Etoposide	0.292	0.01590	0.0902
MON	CFI-400945 (10nM)	Etoposide	0.266	0.00290	0.0010
BT-16		Doxorubicin	1.429	0.00806	
BT-16	CFI-400945 (5nM)	Doxorubicin	0.566	0.00450	0.0118
BT-16	CFI-400945 (10nM)	Doxorubicin	0.576	0.00650	0.0213
BT-16		Etoposide	0.800	0.02269	
BT-16	CFI-400945 (5nM)	Etoposide	0.593	0.01034	0.0743
BT-16	CFI-400945 (10nM)	Etoposide	0.006	0.00873	0.0002
DAOY		Doxorubicin	0.263	0.01178	
DAOY	CFI-400945 (5nM)	Doxorubicin	0.168	0.00309	0.0198
DAOY	CFI-400945 (10nM)	Doxorubicin	0.199	0.00356	0.0573
DAOY		Etoposide	0.495	0.01296	
DAOY	CFI-400945 (5nM)	Etoposide	0.494	0.01744	0.4946
DAOY	CFI-400945 (10nM)	Etoposide	0.800	0.00374	NS

NS - not significant.

^*^IC50 value for combination vs. IC50 control.

### CFI-400945 has potential brain exposure

**Figure 10:**
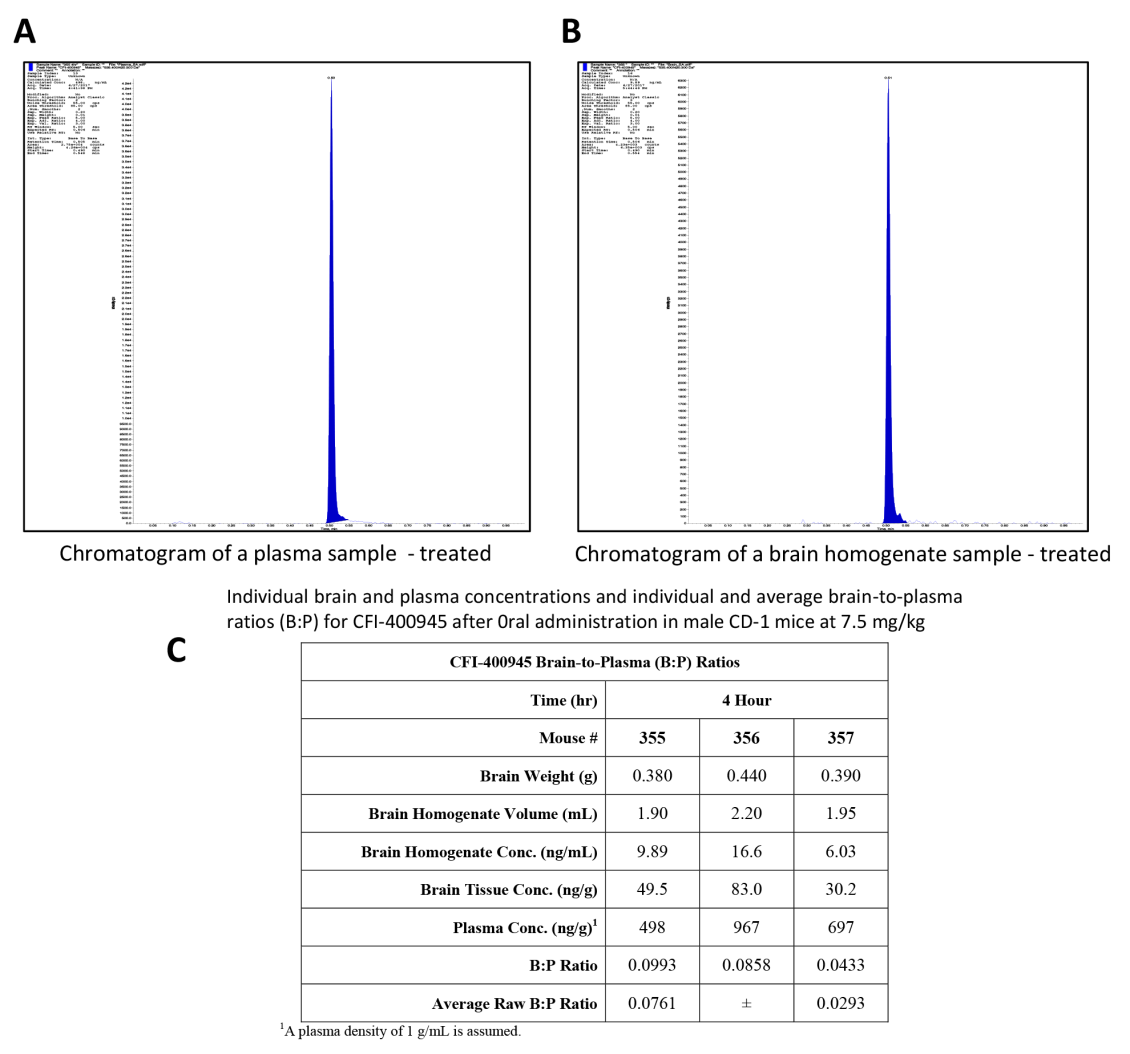
*In vivo* determination of brain-to-plasma ratio (B:P) demonstrated that CFI-400945 penetrates the brain-blood-barrier (BBB) **(A and B)** Pharmacokinetic studies for determination of CFI-400945’s brain-to-plasma ratio (B:P) were performed in fasted male CD-1 mice in triplicates. CFI-400945 was dosed orally (PO) at 7.5 mg/kg/day to the study group but not to the control group. Verapramil was used as internal standard. Blood and brain samples were collected 4 hours post-dose and the concentrations of CFI-400945 in plasma and brain homogenate were determined by LC-MS/MS (Liquid chromatography–mass spectrometry). **(C)** The average B:P for CFI-400945 was 0.0761±0.293.

To determine the potential for blood-brain permeability of CFI-400945, its concentration was measured in plasma and in the brain of male CD-1 mice, using a Tmax based on published pharmacokinetic data [33]. Raw brain-to-plasma ratios (B: P) were determined by dividing the brain concentration in ng/g by the plasma concentration in ng/g (with plasma density assumed to be of 1 g/mL). The average B: P ratio was determined to be approximately 0.08±0.3 (Figure 10). No further CFI-400945 pharmacokinetic studies were done as part of this study.

### The PLK4 inhibitor CFI-400945 produced clinical response in intracranial AT/RT xenografts

**Figure 11:**
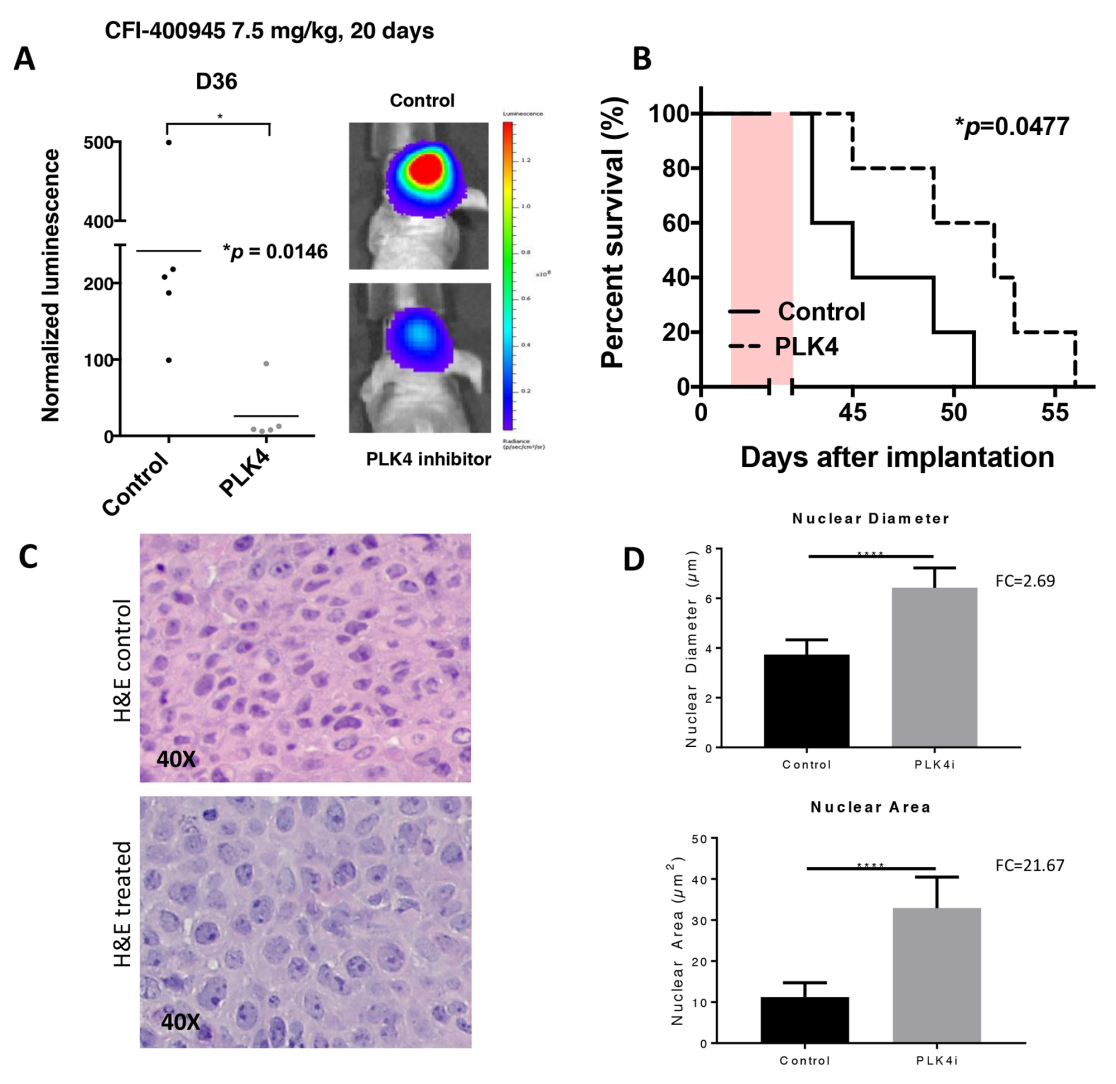
The PLK4i CFI-400945 promoted pharmacological inhibition of AT/RT xenografts Orthotropic xenografts of BT-12 cells treated with CFI-400945 (orally) affected tumor growth and survival. Effects of the compound were also reflected on cell size at histological evaluation. **(A)** Images of AT/RT orthotropic xenografts in mice both control (untreated) and treated with CFI-400945 for 20 days (7.5mg/kg). Mice treated with CFI-400945 displayed decreased luminescence compared to the control (untreated) mice (p=0.0146), indicating that CFI-400945 treatment inhibited tumor growth *in vivo*. **(B)** A survival curve displayed significant impact in survival of mice treated with CFI-400945 compared to the control (untreated) mice (p=0.0477). **(C and D)** Images of H&E stained slides from mice orthotropic xenografts showed significant increase in nuclear diameter (FC=2.69; p-value<0.0001) and nuclear area (FC=21.67; p-value<0.0001) in the tumor cells of the treated mice compared to the control (untreated). *log-rank test.

To determine the *in vivo* anti-tumor activity of CFI-400945, we conducted an experiment in which mice with intracranial AT/RT xenografts were treated with CFI-400945 at 7.5 mg/kg/day consecutively for 21 days by daily oral administration [33]. Treatment with the PLK4 inhibitor significantly reduced the growth of intracranial AT/RT tumors (p=0.0146) and extended the survival of treated animal subjects (p=0.0477). Analysis of brain tumors, obtained from pre-symptomatic mice at the end of the therapy, showed significantly increased nuclear diameter and nuclear area, consistent with CFI-400945 pharmacodynamics (Figure 11).

**Time-lapse video:**
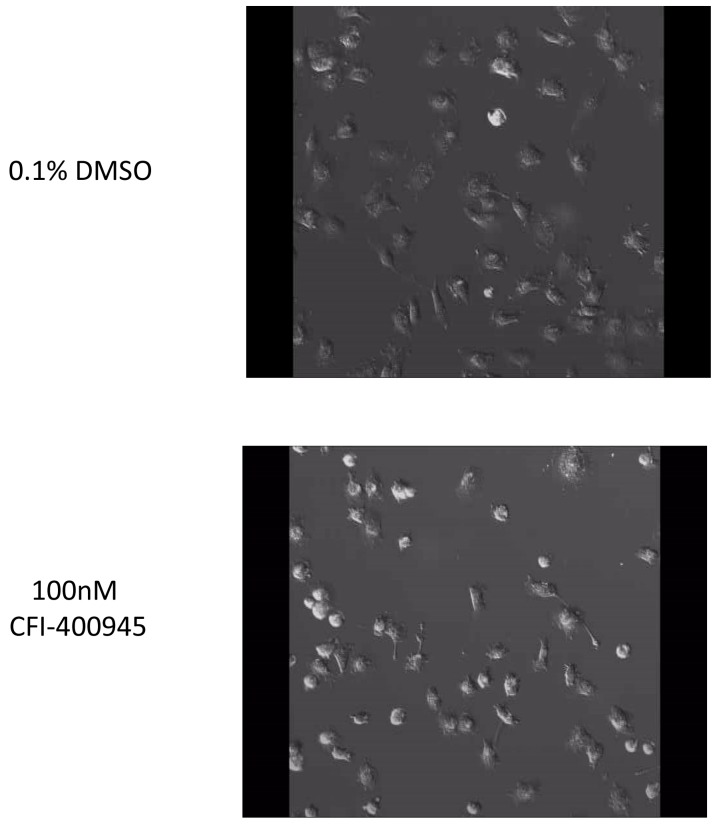
In preparation for mitosis, cells round up detaching from the bottom of the plate In the absence of the inhibitor, cells divide and adhere to the plate. However, in presence of the inhibitor, cells round up, detach and adhere to the bottom of the plate without undergoing cytokinesis. The cells that underwent these unsuccessful mitotic cycles (with DNA endoreduplication without cytokinesis) became hypertrophyc and polyploid. See Time lapse video 1 See Time lapse video 2

## DISCUSSION

We previously demonstrated upregulation of *PLK4* in RT and detected this overexpression also in other embryonal tumors of the brain including pediatric MB [9, 10]. Our preliminary findings indicated that RT cell proliferation was dependent on PLK4 and that the PLK4i CFI-400945 proffered significant therapeutic effects *in vitro*, in RT cells originating from different locations. This suggested that targeting PLK4 with small-molecule inhibitors could represent a novel strategy to treat RT [9]. Here, we further explore the mechanisms of PLK4 inhibition in RT and extend the insights to pediatric MB and *in vivo* effects.

### Protein kinases and kinase inhibitors

Protein kinases regulate multiple cellular functions. They intermediate the coordinated amplification and propagation of cellular stimuli into distinct biological responses via synchronized signal transduction cascades [20]. Mutations and dysregulation of protein-kinases play fundamental roles in human disease, providing the possibility of developing agonists and antagonists for therapeutic purposes. Expanded discovery, research and development of orally active protein-kinase inhibitors has culminated in the approval of many of these drugs for clinical use [34].

### Polo-like kinases

Polo-like kinases (PLKs) are part of the conserved family of serine/threonine kinases that play essential roles in cell cycle progression and DNA damage response. They are often deregulated in cancer [35]. Mammalian cells contain five polo-like family members, PLK 1-5, of which, PLK1 is the most extensively characterized with many inhibitors already in clinical trials [36, 37]. PLK4 is structurally different from the other family members. While PLK1, 2 and 3 possess two Polo-box domains at their C-terminus, PLK4 only has one [14, 38].

### PLK4 activity, regulation and the paradoxical effect of its inhibition

PLK4 activity is essential for centrosome duplication [17]. Centrosomes are present as a single copy at the beginning of the cell cycle and duplicate during S phase producing two copies, each one becoming a spindle pole during mitosis. Within the centrosome, a pair of centrioles controls centrosome duplication [39]. PLK4 is present in its inactive form at centrioles in G1, becoming active in S phase and increasing gradually with progression through the cell cycle. Maximum amount of active PLK4 protein is achieved in mitosis [14]. The *PLK4* gene is transcribed in a cell dependent manner with mRNA expression progressively increasing throughout the cell cycle mirroring the protein levels [14].

PLK4 is a low abundance, short lived protein, with activity regulated by its own stability. This self-regulation occurs through formation of homodimers that trans-autophosphorylate for degradation. This “auto-regulatory feedback loop” [40] keeps PLK4 levels under tight regulation and limits centriole duplication in normal cells [40–42]. Inhibition of PLK4 triggers failure of centriole duplication, while PLK4 overexpression promotes centriole overduplication which results in the formation of extra centrosomes with subsequent genome instability and propensity for tumor formation [43, 44].

It has been demonstrated that Plk4 heterozygous mice are predisposed to tumorigenesis [45]. It has also been established that overexpression of PLK4 promotes centrosome amplification initiating tumorigenesis in flies [46]. Accordingly, Mason and colleagues [11] demonstrated that high dose treatment with CFI-400945 resulted in failure in centriole duplication. Yet, when the authors used lower concentrations of the compound, an increase in centriole number was observed. This bimodal phenomenon or paradoxical effect was also observed in this study when we treated multiple RT and MB cell lines with different concentrations of the inhibitor, as depicted in Figure 4.

This paradoxical effect has been explained by the stable overexpression of kinase-inactive PLK4 upon partial inhibition of PLK4 with low doses of the inhibitor. As a result of this partial inhibition there is formation of heterodimers instead of homodimers. The heterodimers are composed of a kinase-inactive PLK4 molecule and a wild-type PLK4 molecule. In these circumstances, the kinase inactive PLK4 cannot destabilize the active PLK4, leading to an increase in kinase activity. The resultant increase in activity could, in turn, result in higher PLK4 abundance and centriole over-duplication [42]. With high doses of the compound, there is a more extensive inhibition of PLK4 activity that attenuates the feedback of auto-catalyzed destruction. This scenario would result in failure of centriole duplication and consequent blockage of cytokinesis with impairment of cell proliferation [11, 47].

### Polyploidy, senescence and cell death

Polyploidy is defined as increased sets of homologous chromosomes in a cell that might occur due to abnormal cell division. Because an abnormally high chromosome count makes polyploid cells more prone to errors during cell division, polyploidy is considered an undesirable event that may lead to cancer development. However, it has been postulated that polyploid cancer cells are also more sensitive to DNA-damaging agents [27, 29]. In this scenario, induction of polyploidy by inhibiting PLK4 may represent a new approach to cancer therapy.

To induce polyploidy, an agent must allow cells to re-enter a new cycle without going through cytokinesis. We demonstrated that CFI-400945 is an inducer of cancer cell polyploidy in multiple RT and MB cell lines, yielding cell populations with a high degree of polyploidy (>8N) while not affecting non-neoplastic HFF-SCC058 human fibroblasts (Figure 8). These findings are consistent with the phenomenon demonstrated by Tovar and colleagues using the small molecule inducer of polyploidy R1530 where non-cancerous cell were protected from R1530-induced polyploidy [27]. The fact that non-neoplastic proliferating cells seem to be “resistant” to CFI-400945-induced polyploidy supports the rationale for cancer therapy by PLK4 inhibitors.

The mitotic apparatus is frequently unable to cope with a high degree of polyploidy which leads to mitotic catastrophe and cell death [27, 48]. We demonstrated that RT as well as MB cell lines underwent a certain degree of apoptosis during the first 48 hours of exposure to the inhibitor, as illustrated in Figure 5. However, not all polyploid cells underwent apoptosis. A sizeable amount of cells became senescent, remaining viable but not replicating (Figure 6).

Senescence is defined as the permanent loss of proliferative potential of a cell. Senescent cells move from an actively dividing to a non-dividing state, yet remain metabolically active. In conjunction with the loss of the ability to divide, changes occur in cell morphology and in their pattern of gene expression. Although cells may remain viable for a long time, at the end of the process cell death usually occurs [26]. We documented the senescent state of cells treated with CFI-400945 by measuring beta-galactosidase at different time-points. Furthermore, to document the irreversibility of CFI-400945 induced proliferation arrest in RT and MB cells, we detected the loss of the ability to generate colonies after 6 days of exposure to the drug. Although the cells remained alive after 12 days of drug-free media treatment, they did not recover the ability to divide and generate colonies as illustrated in Figure 7.

### Association therapy

Chemotherapy is an important element in current cancer treatments. However, the use of many anticancer drugs is restrained by dose-limiting toxicities as well as development of drug resistance. Therefore, being able to lower the dose of these chemotherapy agents would be highly beneficial for patients, especially children.

Doxorubicin is a potent cytotoxic anthracycline compound and an important part of RT treatment [31, 32, 49, 50]. It intercalates with the DNA and inhibits topoisomerase II. It is highly effective, but its use is limited by cardiotoxicity resulting in dilative cardiomyopathy [51].

Etoposide is also a topoisomerase II inhibitor widely used for cancer therapy. It is a cytotoxic alkaloid compound that causes single or double-strand DNA breaks [52]. It has been used in combination with other agents to treat several embryonal tumors like recurrent MB, high risk Wilms’ tumors, rhabdomyosarcoma, Ewin’s sarcoma and neuroblastoma [53].

It has been postulated that polyploid cancer cells may be more sensitive to DNA-damaging agents [27, 29]. Using viability assays, we demonstrated that the combination of PLK4i CFI-400945 with the conventional chemotherapeutic DNA-damaging agents, doxorubicin or etoposide, resulted in a clear reduction of their IC50 values (Supplementary Table 1 and Figure 9), meaning that induction of polyploidy by the PLK4i CFI-400945 sensitizes RT and MB cell lines to doxorubicin and/or etoposide treatment and therefore, may represent a new approach to cancer therapy.

### Blood brain barrier (BBB) penetration and intracranial xenograft response

Most kinase-targeted drugs that have been investigated, including the PLK4i CFI-400945, were not developed for CNS disorders. The development of kinase-targeted therapies for CNS diseases remains a challenge, and the greatest challenge facing these drugs is the effective penetration of the BBB. It is estimated that only about 2% of small-molecule drugs are able to effectively cross the BBB [54]. The physicochemical properties of a drug considerably influence passive diffusion across biological membranes and the potential to serve as a substrate for the P-glycoprotein (PGP) efflux transporter. Molecular weight (MW), polar surface area (PSA) and lipophilicity (LogP) are key molecular properties that correlate with and may have an important role in influencing the BBB penetrance of a molecule [55]. While CFI-400945 is attractive as an adult stage non-CNS candidate, it is at the cusp of molecular properties (MW=534.65, cLogP=4.48 and PSA=79.48) found in drugs with adequate CNS exposure to allow target engagement. We determined that the brain to plasma ratio (B:P) of CFI-400945 was < 0.1 for CD-1 (“wild type”) mice but did not determine detailed brain pharmacokinetics or screen for brain to plasma ratio in the xenograft model. The most parsimonious explanation for why we were able to observe pharmacodynamics effects in the xenograft tumors consistent with CFI-400945 mechanism of action (and significant cell hypertrophy observed in tumors harvested from treated animals) is the possibility that the BBB is altered in the “disease model state” to make it more accommodating to a less than ideal drug candidate. This is consistent with the concept of BBB physiological changes in disease state that are not evident at the anatomical level [56]. Further, our in *vivo* anti-tumor activity of CFI-400945 was seen at doses previously used by Mason and colleagues to treat peripheral xenograft tumors [11]. Clearly, there are other likely explanations, such as retention or deposit of CFI-400945 in brain tissue after repeated daily dosing over an extended period of time. However, these various theoretical possibilities were not pursued as part of this study. The key relevant aspect to our overall goal was the demonstration that CFI-400945 treatment significantly reduced growth of intracranial tumors and significantly extended the survival of treated animals. Future screens for CNS therapeutic candidates or refinement of existing peripheral tissue therapeutics will be needed to address the brain exposure challenge in order to optimize candidates for future clinical trials.

### Kinase selectivity and risk potential for PLK4 inhibitors

All FDA approved kinase inhibitor drugs are multi-kinase inhibitors and approvals are directed by oncology indications. Further, the approved kinase inhibitor drugs are dominated by those that target the tyrosine protein kinase (Y-PK) family. Only four appear to target the serine/threonine protein kinase (S/T-PK) class as their molecular mechanism of action. None of the approved protein kinase inhibitor drugs are for CNS indications. PLK4 is an S/T-PK, and we are addressing brain tumors in our studies. Therefore, the finding that a prototype PLK4 inhibitor drug candidate, CFI-400945, is effective in a brain tumor model is a significant finding in itself. The additional finding that CFI-400945 falls within the norm of approved oncology therapeutics that are typically multi-kinase inhibitors with a subset of kinome selectivity is encouraging. As with extant approved kinase inhibitor drugs, some of the cross-over kinase hits for CFI-400945 under 1,000 nM IC50 are not unexpected based on kinase structural similarities (e.g., aurora kinases) and others fit the prevailing view that a set of multiple kinases must be hit for optimal efficacy [22]. Further probing of kinase inhibitor specificity links to *in vivo* efficacy must await the availability of more selective kinase inhibitors with CNS exposure properties. Our results with CFI-400945 in brain tumor models indicate the potential therapeutic utility of such endeavors. Finally, the cardiotoxicity associated with cancer therapeutics and Y-PK inhibitor drugs does not appear to be a primary concern for PLK4 targeting with mixed kinase inhibitors as represented by CFI-400945, nor is there an obvious high pharmacogenonomic risk for individual variability in response or toxicity based on CYP2D6 activity.

## MATERIALS AND METHODS

### Ethics statement

Investigation has been conducted in accordance with the ethical standards and according to the Declaration of Helsinki and according to national and international guidelines and has been approved by the authors’ institutional review board.

### Cell culture

MON cells, which were provided by Dr. Delattre (Institute Curie, Paris, France), were established from an abdominal rhabdoid tumor [57–59]. The G401 cell line (ATCC, USA) was established from a RTK. The AT/RT cell lines, BT-12 and BT-16, which have been extensively used in preclinical studies [60, 61], were established by Drs. Houghton and Biegel (Nationwide Children’s Hospital, Columbus, Ohio and The Children’s Hospital of Philadelphia, Philadelphia, Pennsylvania, respectively). The two MB cell lines DAOY and D283 (ATCC, USA) are classified as belonging to the sonic hedgehog (SHH) molecular subgroup and group 3 / 4 respectively [21]. As a non-neoplastic control, we used HFF-SCC058 cells (EDM Millipore, USA) derived from human foreskin fibroblasts which have significantly lower expression of PLK4 [9]. MON, BT-12 and BT-16 cells were maintained in HyClone RPMI 1640 (GE Healthcare Life Sciences, USA), G401 in McCoy’s 5A (Sigma Aldrich, USA), HFF-SCC058 in DMEM (Thermo Fisher Scientific, USA), and DAOY and D283 in EMEM (Thermo Fisher Scientific, USA). All media were supplemented with 10% FBS and 1% penicillin/streptomycin and cells were maintained at 37°C, 5% CO_2_.

### Kinase activity screening

The inhibitory activity of CFI-400945 on various kinases was assessed through 486 biochemical kinase assays using 500 nM of the compound. These assays utilized various formats appropriate to the kinase, its substrate and its activity. Formats included the Z-LYTE™ Kinase Assay that determines the differential sensitivity of phosphorylated and non-phosphorylated peptide substrates to proteolytic cleavage using a FRET-based readout, the Adapta™ Universal Kinase Assay that employs a homogeneous, fluorescence-based immunoassay for the detection of ADP produced by kinases and the LanthaScreen™ Eu Kinase Binding Assay that utilizes an Alexa Fluor™ conjugated “tracer” and an Eu-labeled anti-tag antibody to measure binding of a compound to the kinase target [62]. The half maximal inhibitory concentration (IC50) at selected, highly inhibited kinases was further evaluated by 10-point titration (SelectScreen™ Kinase Profiling Services - Thermo Fisher Scientific, USA) [63, 64].

### Drug safety and toxicology

Analysis of CFI-400945 against a panel of P450s isoenzymes (1A2, 2B6, 2C8, 2C9, 2C19, 2D6, 2J2, 3A4 and 3A5) and Vivid™ substrates was determined using 10-point titrations, in duplicates to determine IC50 values (SelectScreen™ P450 Profiling Service – Thermo Fisher, USA) [65, 66]. To determine if the compound binds the cardiac hERG channel, the IC50 was quantitated using a 10-point titration, in duplicates in a hERG fluorescence polarization assay (SelectScreen™ hERG Screening Service – Thermo Fisher Scientific, USA) [67].

### Cell proliferation, colony formation, migration and invasion assays of MB cell lines

Cell proliferation, colony formation, migration and invasion of the MB cell lines DAOY (SHH subtype) and D283 (group 3/4 subtype) were performed as previously described [9]. For proliferation assay DAOY was plated with 5 x 10^4^ cells per well and D283 was plated with 5 x 10^5^ cells per well.

### Compounds

Cells were treated as indicated below with the following compounds: CFI-400945 (CAS 1338800-06-8 – Cayman Chemical, USA), doxorubicin (CAS 25316-40-9 – MedChem Express, USA) and etoposide (CAS 33419-42-0 – Cayman Chemical, USA).

### Viability assay

To evaluate cell viability, we used the Presto Blue™ Cell Viability reagent (Thermo Fisher Scientific, USA). Cells were plated in triplicates in 96-well plates. Except DAOY and D283, all cell lines were plated with 2 x 10^3^ cells per well 96-well and the absorbance was measured after 48 and 72 hours at drug concentrations of 5, 10, 50, 100, 200, 500nM, 1μM and 5μM. DAOY was plated with 5 x 10^4^ cells per well and D283 was plated with 5 x 10^5^ cells per well.

### Confluency assay

For further evaluation of the effect of the PLK4 inhibitor over tumor cell proliferation, confluency was measured over a 48 hour period. MON, BT-12, G401 and DAOY cells were plated in 96-well plates with a density of 6 x 10^4^ per well. On day 1, CFI-400945 was added to the cells in increasing concentrations (0, 0.12, 0.49, 1.95, 7.81, 31.25, 125 and 500 nM). Confluency was monitored for 48 hours by taking phase contrast images every 2 hours and measured using an IncuCyte ZOOM system (Essen BioScience, USA). Experiments were performed in triplicates.

### Immunofluorescent assays

Cells treated with various concentrations of CFI-400945 and 0.1% DMSO (control) were seeded on Poly Lysine (Sigma, USA) coated coverslips, washed twice with 1X PBS, fixed in ice cold Methanol (Fisher Chemical, USA) at -20°C for 10 min and then permeabilized with 0.25% Triton-X (Acros Organics, USA) at room temperature (RT) for 10 min. The cells were then blocked in blocking buffer (5% BSA (Jackson Immuno Research Laboratories Inc., USA) in PBST) for 1 hour at RT. The primary antibody (Anti-λ Tubulin 1:500, Sigma, USA) diluted in 1% BSA in PBST was then added and incubated at 4°C overnight. Then, the cells were washed with 1X PBS thrice. Secondary antibody (Anti-Rabbit IgG (H+L) F (ab’) 2, 1:500, Sigma, USA) diluted in 1% BSA in PBST was added and incubated at RT for 2 hours. Three time washes with 1X PBS were performed. Then, 1mL of DAPI (Thermo Fisher Scientific, USA) in PBS (0.5μl of DAPI in 10 ml PBS) was added to the cells and incubated for 10 min at RT. Finally, 3 time washes with 1X PBS were performed and slides were mounted using Mounting Medium (Thermo Fisher Scientific, USA).

### Flow cytometry - apoptosis

Cells were treated with various concentrations of CFI-400945 and 0.1% DMSO (control) for evaluation of the impact of treatment on induction of apoptosis. Live cells were stained with Annexin V as an indicator of early apoptosis and Propidium Iodide (PI - Thermo Fisher Scientific, USA) for detection of late apoptosis and necrosis, according to manufacturer’s instructions (Thermo Fisher Scientific, USA). Flow cytometric analysis was performed on a BD Fortessa instrument (BD Biosciences, USA) within an hour of staining. Data was analyzed using Modfit LT from Verity Software House. All experiments were performed in triplicates.

### Flow cytometry - cell cycle

Cell cycle evaluation was performed by staining the cells with PI (Thermo Fisher Scientific, USA) according to manufacturer’s instructions. Cells treated with CFI-400945 and 0.1% DMSO (control) were fixed in 80% ethanol overnight, stained with PI and then subjected to flow cytometric analysis using a BD Fortessa instrument (BD Biosciences, USA). Data was analyzed using Modfit LT from Verity Software House. Experiments were performed in triplicates.

### Senescence

Senescence of cells treated with CFI-400945 and 0.1% DMSO (control) was detected using the Senescence Cells Histochemical Kit (Sigma, USA), which is based on a histochemical stain for beta-galactosidase activity at pH 6. For each cell line, cells were seeded in 6-well plates in triplicates with 100,000 cells per well. Treatment medium was added the next day and incubated for 48 or 72 hrs. After incubation, cells were washed twice with 1X PBS and 1mL of the Fixation Buffer from the kit was added to each well of the plate and incubated at RT for 5 min. Cells were washed with 1X PBS and 1mL of staining solution from the kit was added to each well and incubated at 37°C without CO_2_ overnight. The next day, the cells were washed with 1X PBS and the numbers of blue stained cells (which indicate senescent cells) and unstained cells were counted in image J (www.imagej.nih.gov). The percentage of senescent cells was calculated.

### Clonogenic recovery assay

For each cell line, 200 cells were seeded into 6-well tissue culture plates. Cells were incubated for 6 days with 100nM of CFI-400945. On the 7^th^ day, cell were washed with 1X PBS twice and then incubated with 0.1% DMSO for an additional 12 days. Controls were: (1) incubated with 0.1% DMSO for 18 days and (2) incubated with CFI-400945 100nM for 18 days. The number of colonies was counted. All experiments were performed in triplicates.

### Quantitative real-time PCR

The expression of *PLK4* (Hs00179514_m1) in each cell line was verified by TaqMan GE assays (Thermo Fisher Scientific, USA) using the housekeeping gene *GAPDH* (Hs02758991_g1) as reference. Total RNA (2μg) was used to make cDNA using the Applied Biosystems High Capacity RNA-to-cDNA kit (Thermo Fisher Scientific). Reactions were performed in triplicate with adequate positive and negative controls. The normalized expression levels were calculated by the ΔΔCt method using the housekeeping gene and a pool of all samples as calibrator.

### Time-lapse assay

Time-lapse images of MON cells treated 100nM CFI-400945 and 0.1% DMSO for 48 hours were taken using a Zeiss LSM 800 confocal microscope (Zeiss XL-LSM710S1 live-cell incubator system with CO_2_ Module S, and Heating Unit XL S, Zeiss, USA) set to normal growing conditions (37°C, 5% CO_2_). Cells were plated in 35mm glass bottom dishes (Cat#150682, Thermo Fisher Scientific, USA) in normal growth medium and allowed to grow overnight. After the treatment medium was added, imaging began immediately. Brightfield images (20X) were taken every 6 minutes at 10 predetermine fields for 48 hours. Images were processed in ZEN 2.3 software (Zeiss, USA).

### Combination therapy - viability assay

To quantify the effects of the treatment with the PLK4i CFI-400945 in combination with DNA-damaging chemotherapy agents, viability assays were performed using the Presto Blue™ Cell Viability reagent (Thermo Fisher Scientific, USA). Cells were plated in triplicates in 96-well plates. Cells were treated with a combination of 5 or 10nM CFI-400945 and 1, 5, 10, 50, 100, 200, 500nM, 1 and 5 μM of doxorubicin or etoposide for 72 hours. Cells were also treated with the same concentrations of CFI-400945, doxorubicin and etoposide alone, as well as 0.1% DMSO as controls. The percentage of viable cells compared to the controls was calculated using the average DMSO absorbance values compared to the treated absorbance values. The percent of viable cells’ curves were made using the statistical software PRISM (GraphPad Software, USA) and IC50 values were calculated from the curves. P-values were calculated by (unpaired *t*-test) comparing the absorbance values of the combination therapy and each cytotoxic drug alone at the nearest IC50 value of the combination therapy.

### Brain to plasma ratio (B:P)

Male CD-1 mice weighing between 20 and 40 grams were fitted with jugular vein cannulas as appropriate and fasted overnight until 4 hours post-dose. Double route of administration (oral and IV) were tested, with a single dose concentration of 7.5mg/kg [11]. Plasma and brain were sampled at 6 time-points, starting at 30 minutes (n=3 animals/time-point). Brain was extracted at the same plasma sampling time-point from each animal. Both plasma and brain samples were deproteinated. Brain samples were weighted, homogenized and CFI-400945 was extracted. Then, mass spectrometry (MS) optimization for CFI-400945 detection was performed; liquid chromatography (LC) evaluation using a generic reverse phase column and gradient method for separation of CFI-400945 from matrix interference was performed. Finally, a 6 point standard curve for CFI-400945 with an internal standard (Verapamil) and determination of CFI-400945 concentrations in the dosing solutions and incurred samples was performed using a generic LC-MS/MS method. The dosing solution was normalized in a matched matrix (mouse plasma) and analyzed in triplicate in the same analytical batch as the incurred samples (Absorption Systems - Exton, PA).

### Intracranial xenograft tumors

Six-week-old female athymic mice (rnu/rnu genotype, BALB/c background) were purchased from Envigo (Harlan) and housed under aseptic conditions, which included filtered air and sterilized food, water, bedding, and cages. The Northwestern University Institutional Animal Care and Use Committee approved all animal protocols. The BT-12 AT/RT cell line, which has high expression of PLK4 was modified to express luciferase and injected intracranially in nude mice as previously described [68]. Each mouse was injected with a cell suspension (10,000 cells/3μL) into the right caudate putamen of the brain. Mice injected BT-12 cells (n=20) were randomized in two treatment groups: 1) control (vehicle alone), 2) CFI-400945 (7.5 mg/kg/day for 21 days). Bioluminescence monitoring was performed twice a week throughout the course of the experiment for quantitative measurement of tumor growth and response to therapy. Kaplan-Meier survival analysis was performed to assess survival benefit within the treatment group as compared to controls. Differences between survival curves were calculated using a log-rank test. Brains were resected at 6 hours following completion of the treatment and placed in 4% paraformaldehyde for subsequent histological examination.

## CONCLUSION

We demonstrated initial evidence of PLK4 as a CNS druggable target for rhabdoid tumors and MB. Our findings indicate that targeting PLK4 with optimized small-molecule inhibitors may hold a novel strategy for the treatment of embryonal tumors, including those of the CNS.

## SUPPLEMENTARY MATERIALS TABLE




