# Supplementary material for: Inhibition of polo-like kinase 4 (PLK4): a new therapeutic option for rhabdoid tumors and pediatric medulloblastoma
Source: Oncotarget. 2017 Nov 24;8(67):111190–212. doi: 10.18632/oncotarget.22704 (PMC5762315; doi:10.18632/oncotarget.22704)
Supplement: Supplementary file 3 [file oncotarget-08-111190-s001.pdf]

Inhibition of polo-like kinase 4 (PLK4): a new therapeutic option for rhabdoid tumors and pediatric medulloblastoma

SUPPLEMENTARY MATERIALS

Supplementary Table 1: CFI-400945 – Kinase screening.

See Supplementary File 1
